# The value of a dual-energy CT Iodine map radiomics model for the prediction of collagen fiber content in the ccRCC tumor microenvironment

**DOI:** 10.1186/s12880-023-01127-x

**Published:** 2023-11-15

**Authors:** Zhongyuan Li, Ning Wang, Xue Bing, Yuhan Li, Jian Yao, Ruobing Li, Aimei Ouyang

**Affiliations:** 1 School of Medical Imaging, Weifang Medical University, No. 7166, Baotong West Street, Weifang, Shandong 261053 P. R. China; 2 Department of Radiology, Central Hospital Affiliated to Shandong First Medical University, No. 105 Jiefang Road, Jinan, Shandong 250013 P. R. China; 3 Department of Radiology, Longkou Traditional Chinese Medical Hospital, Yantai, Shandong 265700 P. R. China; 4 Shandong First Medical University, No. 105 Jiefang Road, Jinan, Shandong 250013 P. R. China

**Keywords:** Renal cell carcinoma, Tumor microenvironment, Collagen fibers, Dual-energy CT, Radiomics; machine learning

## Abstract

**Background and purpose:**

Renal cell carcinoma (RCC) is a heterogeneous group of cancers. The collagen fiber content in the tumor microenvironment of renal cancer has an important role in tumor progression and prognosis. A radiomics model was developed from dual-energy CT iodine maps to assess collagen fiber content in the tumor microenvironment of ccRCC.

**Methods:**

A total of 87 patients with ccRCC admitted to our hospital were included in this retrospective study. Among them, 59 cases contained large amounts of collagen fibers and 28 cases contained a small amount of collagen fibers. We established a radiomics model using preoperative dual-energy CT scan Iodine map (IV) imaging to distinguish patients with multiple collagen fibers from those with few collagen fibers in the tumor microenvironment of ccRCC. We extracted features from dual-energy CT Iodine map images to evaluate the effects of six classifiers, namely k-nearest neighbor (KNN), support vector machine (SVM), extreme gradient boosting (XGBoost), random forest (RF), logistic regression (LR), and decision tree (DT). The effects of the models built based on the dynamic and venous phases are also compared. Model performance was evaluated using quintuple cross-validation and area under the receiver operating characteristic curve (AUC). In addition, a clinical model was developed to assess the clinical factors affecting collagen fiber content.

**Results:**

Compared to KNN, SVM, and LR classifiers, RF, DT, and XGBoost classifiers trained with higher AUC values, with training sets of 0.997, 1.0, and 1.0, respectively. In the validation set, the highest AUC was found in the SVM classifier with a size of 0.722. In the comparative test of the active and intravenous phase models, the SVM classifier had the best effect with its validation set AUC of 0.698 and 0.741. In addition, there was a statistically significant effect of patient age and maximum tumor diameter on the collagen fiber content in the tumor microenvironment of kidney cancer.

**Conclusion:**

Radionics features based on preoperative dual-energy CT IV can be used to predict the amount of collagen fibers in the tumor microenvironment of renal cancer. This study better informs clinical prognosis and patient management. Iodograms may add additional value to dual-energy CTs.

## Introduction

Renal cell carcinoma (RCC) is a heterogeneous group of cancers that includes many histological subtypes, of which clear cell histology is the most common subtype [1]. RCC is the seventh most common cancer-causing death worldwide, with 140,000 patients dying from this cancer each year [2–4]. The tumor microenvironment (TME) is the environment in which tumor cells grow and develop, which includes not only the tumor cells themselves but also surrounding cells such as immune cells, fibroblasts, and glial cells, as well as collagen fibers, interstitial cells, microvasculature and other biomolecules surrounding the tumor. The presence of these cells is critical to tumor development, treatment, and prognosis. In recent years, tremendous progress has been made in tumor cytology and molecular biology, and researchers have gained a deeper understanding of the relationship between tumors and their environment. These results not only help us understand tumorigenesis, development, and metastasis but also help us better diagnose, prevent and treat tumors. Tumor cells can interact with surrounding cells through multiple pathways, thus influencing the development and progression of cancer. In addition, non-malignant cells in the tumor microenvironment play a key role in all stages of cancer development by stimulating cell proliferation. TME is a complex spatial network of interwoven extracellular matrix proteins, of which collagen is a major component of the ECM, and several studies have shown that abnormal aggregation of collagen fibers is highly correlated with tumor progression [5–7]. In addition, the fiber component also promotes the growth, infiltration, and distant metastasis of ccRCC cells and therefore plays a crucial role in the growth of ccRCC. Assessing the fiber content of the tumor microenvironment in ccRCC patients is essential for understanding tumor cell progression and prognostic treatment.

CT scan is commonly used as the primary imaging technique to detect kidney cancer. Conventional CT cannot accurately assess primary tumors due to its relatively low soft tissue resolution. Dual-energy CT imaging refers to the acquisition of data from two different energies of electrons through a single scan [8]. It has been pointed out that dual-source CT dual-energy scanning can solve the problem of conventional CT scans with more single data, and virtual flat-scan images as well as Iodine maps are obtained after corresponding software processing, and the advantage of this technique is that it can reduce motion artifacts and improve resolution [9]. Li et al. [10] improved the ability to identify tissue enhancement by comparing lesions' density and change characteristics on the IV when evaluating the contrast uptake of lesions. Ideograms allow for both qualitative analysis of iodine content and quantitative analysis of iodine concentration, and this post-processing technique provides useful information for differential diagnosis.

The concept of radiomics was formally introduced in 2012, and in recent years it has shown excellent performance in oncology applications. Currently, the application of radiomics in kidney cancer is focused on three aspects: ( 1) differentiation of benign and malignant renal tumors [11, 12]; (2) staging and grading [13, 14]; and (3) differentiation of different subtypes of kidney cancer [11, 15]. The application of radiomics with dual-energy CT IV imaging to assess the fibrous component in renal cell carcinoma is rare. This study aimed to develop radiomics models from dual-energy CT reconstructed Iodine map images for preoperative prediction of fibrous component content in patients with ccRCC, to improve the treatment and prognosis of patients with ccRCC. In this study, we investigated the feasibility of applying radiomics based on dual-energy CT iodine concentration images to assess the fibrous component content in renal cell carcinoma by combining clinical data (age, gender, maximum tumor diameter, nuclear grading, stage, presence of envelope and presence of necrotic areas).

## Patients and methods

### Study cohort

This study was a retrospective study that was approved by the Ethics Committee of Shandong Qilu Hospital, whose ethics committee waived the requirement of informed consent. The study included 87 patients (60 males and 27 females, with a mean age of 57 years ± 10.94 years, ranging from 33 to 82 years) admitted to Shandong Qilu Hospital. Inclusion criteria were: (1) patients with pathologically confirmed RCC after partial or radical nephrectomy; (2) patients who obtained a complete dual-energy CT scan preoperatively. Exclusion criteria were: (1) patients with non-clear cell carcinoma; (2) patients receiving pre-operative radiotherapy and targeted therapy; and (3) patients with poor imaging due to other diseases including cardiac, hepatic, and renal insufficiency. We used the Radcloud Radiomics Platform (Huiying Medical Technology Co, https://mics.huiyihuiying.com/) to manage the imaging data, clinical data, and subsequent statistical analysis of radiomics. The validation and training datasets were separated using a randomized method with a ratio of 3:7 and a random seed of 468 (Examples of groupings are shown in Table 1).

**Table 1:** Grouping of DT classifiers

Clinical characteristics	Training (*n* = 60)	Validation (*n* = 27)	Chi-square value	*P*-Value
Mean age (year)	56.55	58.37	0.506	0.479
Gender (n)			0.036	0.529
Male	41	19		
Female	19	8		
Amounts of collagen fibers (n)			0.242	0.399
Large	41	17		
Small	19	10		
Mean maximum tumor diameter (cm)	4.688	4.459	0.224	0.637
Nuclear grading (n)			0.645	0.293
High	13	8		
Low	47	19		
Tumor stage (n)			5.175	0.159
I	36	21		
II	6	3		
III	9	3		
IV	9	0		
Envelope (n)			0.074	0.483
Presence	33	14		
Absence	27	13		
Necrotic areas (n)			< 0.001	0.601
Presence	40	18		
Absence	20	9		

### Clinical data

All patient data included in this study were collected from our hospital's integrated electronic medical record system. The clinical data included age, gender, maximum tumor diameter, nuclear grading, stage, envelope presence, and necrotic areas.

### Study methods

#### CT scan method

The patient is flexed laterally, lying flat and resting, and is fixed in the middle of the scanning bed. The patient's toes are advanced laterally and the internal pressure line is positioned at the sternocarpal process and the horizontal line is positioned at the mid-axillary line. The contrast agent concentration (iohexol, 300 mg/ml) was 60 ~ 80 ml, which was injected by a high-pressure syringe into the patient's forearm vein, with an average intravenous flow rate of about 3 ~ 5 ml/s per minute. Then a double-phase enhancement scan of the dermatomedullary and parenchymal phases was performed in dual-energy mode. The delayed exposure time was adjustable to 30 and 80 s, meanwhile, the tube flow voltage was adjustable to Sn100kVp and Sn150kVp. Adjustable tube flows a current of 130-180mAs and 80-90mAs with automatic delayed exposure system on.

#### Pathological evaluation

**Fig. 1:**
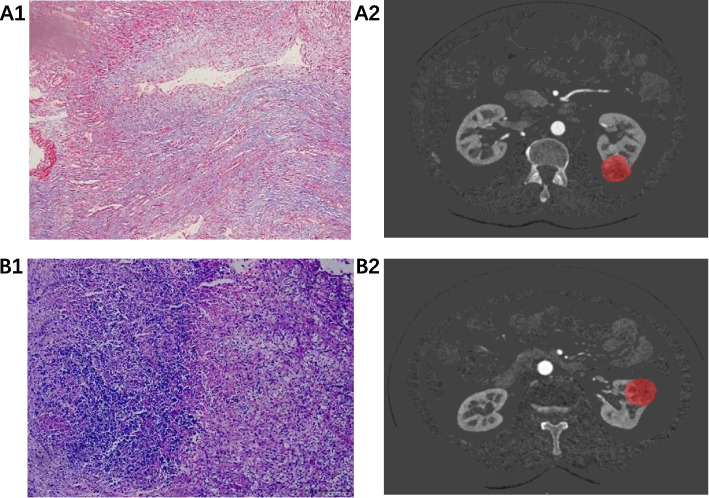
Masson stained pathological sections [The collagen fiber composition is shown after Masson staining (magnification × 200), and the blue area is collagen fiber.] and corresponding CT iodogram images. Negatives: A1 ~ 2 a 56-year-old male with biopsy-confirmed renal carcinoma clear cell carcinoma a large amount of collagen fibers; B1 ~ 2 a 63-year-old female with biopsy-confirmed renal carcinoma clear cell carcinoma with a small amount of collagen fibers

Samples are taken from the tumor site of the wax block. The tissue was cut into sections of 4 µm thickness, and paraffin-sealed slices were made. Then patient sections were stained for Masson histopathology. Pathologist with 5 years of experience performing observations under a light microscope. Based on the pathological findings, they were divided into two groups: a large amount of collagen fibers and a small amount of collagen fibers. At the hotspot for collagen fibers, a small amount was designated if it covered less than half of the visual field, while a large amount referred to coverage greater than this threshold (Fig. 1).

#### Tumor segmentation

The images were uploaded to the Radcloud Radiomics Platform (http://mics.radcloud.cn/), and two radiologists with more than 1–2 years of experience manually outlined the entire lesion layer by layer on all consecutive levels of the tumor, and then radiologists with more than 5 years of experience reviewed all the contours, and if the discrepancy was ≥ 5%, the senior radiologist determined that the tumor boundary as [16]. After a week, 30 cases were randomly selected, and the two radiologists repeated the segmentation) to assess the inter- and intra- class correlation coefficients (ICCs). ICC > 0.75 was regarded as favorable robustness and reproducibility. We did a physician consistency check and found that there was good consistency in the division of the ROIs between the two physicians.

#### Feature extraction and selection

Two thousand eight hundred eighteen quantitative imaging features were extracted from CT images by the Radcloud platform (http://radcloud.cn/), including 1409 features each in the arterial and venous phases. These features can be divided into three groups. The first category (first-order statistics) includes 252 descriptors that quantify the distribution of voxel intensities in CT images using commonly used basic metrics. The second category (shape and size-based features) includes 28 3D features that reflect the region's shape and size. Based on the gray run length and gray co-occurrence texture matrix calculations, 1050 texture features that can quantify the heterogeneous differences of the region are grouped into the third group (texture features). In summary, a large number of image features can be computed. However, all these extracted features may not be useful for a specific task. Therefore, downscaling and selecting task-specific features for optimal performance are necessary steps. Feature selection methods that reduce redundant features include variance thresholding (variance threshold = 0.8), SelectKBest, and the least absolute shrinkage selection operator (LASSO). Since the threshold value of the variance threshold method is 0.8, the feature values with variance less than 0.8 are removed. The select best method is a univariate feature selection method that analyzes the relationship between features and classification results using *p*-values, as all features with *p*-values less than 0.05 are used. The L1 regularizer is used as the cost function in the LASSO model, the cross-validation error value is 3, and the maximum number of iterations is 1000.

#### Machine learning model building

After feature characterization, a total of 2818 features were identified as significantly related to the topic. Based on the selected features, the radiomics score (Rad-score) was computed individually for each patient through a mathematical formula that incorporates a selection of $n$ radiomics signatures. The Rad-score formula was derived as follows: Rad-score = $\alpha +n{\sum }_{i=1}^{n}\beta iXi$, and $\alpha$ represents the intercept, $\beta i$ denotes the value of radiomics feature; $Xi$ represents the corresponding coefficient.

Several supervised learning classifiers are available for classification analysis, which create models that attempt to separate or predict data related to outcomes or phenotypes (e.g., patient outcomes or responses). In this study, six classifiers, k-nearest neighbor (KNN), support vector machine (SVM), extreme gradient boost (XGBoost), random forest (RF), logistic regression (LR), and decision tree (DT), were used to construct radiomics-based models, and validation methods were used to improve the validity of the models and to compare the variability and superiority of the kinetic-venous phase models.

#### Statistical methods

Statistical analysis was performed on the Radcloud platform. In the training and validation datasets, we used subject operating characteristic (ROC) curves, or area under the curve (AUC), to assess the performance of the predictions. Four metrics, P [accuracy = true positives/(true positives + false positives)], R [check-all rate = true positives/(true positives + false negatives)], f1-score [f1-score = P*R*2/ (P + R)], and support (total number of test sets), were employed in this study to assess how well the classifiers performed.

SPSS 25.0 (IBM) was used to analyze the above data statistically, and differences were considered statistically significant at *P* < 0.05. The clinical data were analyzed by chi-square test and multiparametric regression analysis. All statistical tests were performed using one-sided and two-sided tests.

## Results

### Clinical characteristics

A total of 87 patients (60 males as well as 27 females; mean age 57 years ± 10.94; age range 33 to 82 years) were included in this study, and the number of patients with multiple collagen fibers was 59 (68.0% of cases) and 28 (32.0% of cases) with small amount collagen fibers. The maximum diameter of the tumor was obtained on the transverse axis of the image images, and its size ranged from 1.2 cm to 11.0 cm. In the training cohort, there were significant differences (*P* < 0.05) in patient age and tumor maximum diameter between the content of multiple collagen fibers and the content of small amount of collagen fibers (Tables 2 and 3), and the rest of the gender, nuclear grading, tumor stage, presence or absence of envelope and presence or absence of necrotic areas were not significant (*P* > 0.05) (Table 4).

**Table 2:** Relationship between patient age, maximum tumor diameter, and collagen fiber content

Effect	Model fitting conditions	Likelihood ratio test		
	-2 log likelihood of the simplified model	chi-square test	Degree of freedom	Significance
Intercept	6.945^a^	.000	0	
Age	54.938	47.993	29	.015
Maximum tumor diameter	69.986	63.041	41	.015

The chi-square statistic is the difference in -2 log likelihood between the final model and the simplified model. The simplified model is formed by omitting an effect from the final model. The original assumption is that all parameters of the effect are 0

^a^Since omitting this effect does not increase the degrees of freedom, this simplified model is equivalent to the final model

**Table 3:** Relationship between patient age and maximum tumor diameter and collagen fiber content

Model	Model fitting conditions	Likelihood ratio test		
	-2 log likelihood of the simplified model	chi-square test	Degree of freedom	Significance
Intercept distance only	110.172			
The final	6.945	103.227	75	.017

**Table 4:** Relationship between patients' clinical characteristics and collagen fibre content (*p*-value) (*n* = 87)

Clinical Characteristics	Large (*n* = 59)	Small (*n* = 28)	Total (*n* = 87)	Chi-square value	*P*-Value
Gender				0.421	0.346
Male	38	21	59		
Female	20	8	28		
Nuclear Grading				1.130	0.215
High	16	5	21		
Low	42	24	66		
Tumor Stage				0.629	0.890
I	36	21	57		
II	6	3	9		
III	9	3	12		
IV	6	3	9		
Envelope				0.023	0.530
Presence	31	15	46		
Absence	27	14	41		
Necrotic Areas				0.026	0.528
Presence	39	19	58		
Absence	19	10	29		

### Classification results

**Fig. 2:**
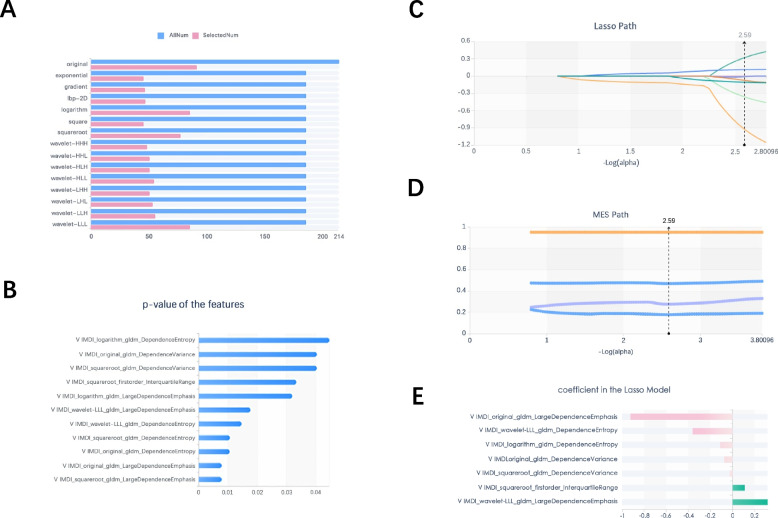
**A** 892 features were selected from 2818 using the variance threshold method (variance threshold = 0.8).** B** 11 features were further selected using the best method Select K. **C** ~ **E** 7 features corresponding to the best alpha values were selected using the Lasso model

Radiomic features with good intra- and interobserver reproducibility (ICC > 0.75) were selected from all 2818. Then we filtered 892 features from the features using the variance threshold method, then 11 features using the best selection method K. Finally, 7 optimal features were screened by the LASSO algorithm (Table 5 and Fig. 2). Using the same screening method, 444 and 445 features were screened out of 1409 features in the arterial and venous phases, respectively, and the best method K was selected to screen 3 and 4 features, respectively, and the optimal features were both screened as 3 using the LASSO algorithm (Table 6).

**Table 5:**
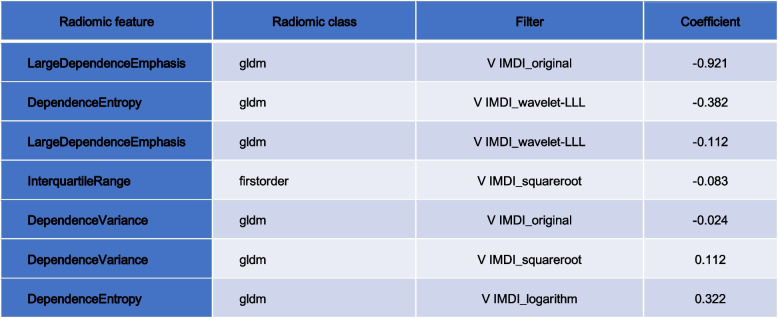
Description of the selected radiomics features with their associated feature group and filter

Label: GLDM = Gray Level Dependence Matrix

**Table 6:**
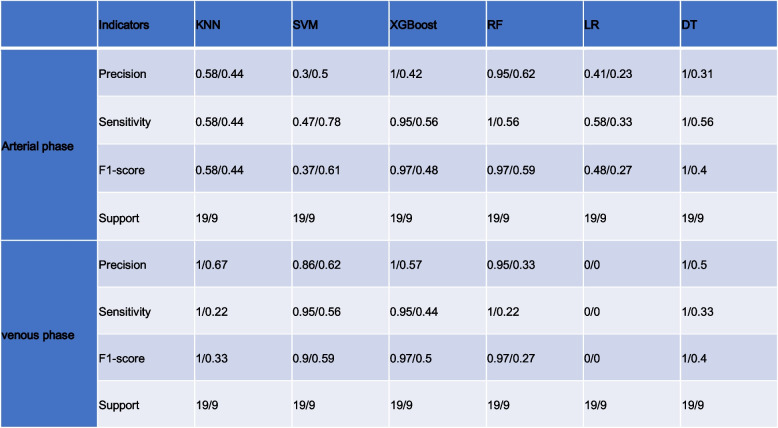
The results of Precision, Sensitivity, F1-score, and Support in training/validation cohorts of the arterial phase and venous phase

**Fig. 3:**
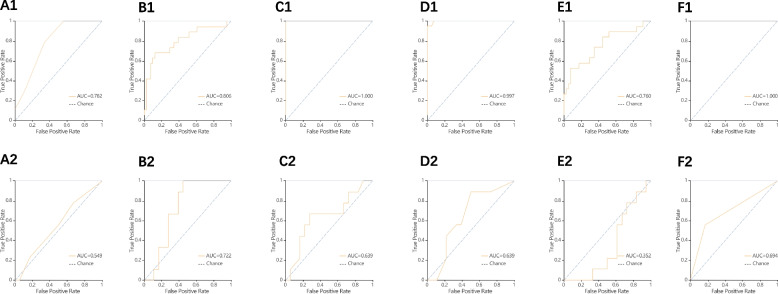
ROC curves of six methods (KNN, SVM, XGBoost, RF, LR and DT). A1 ~ F1 ROC curves for the training set in a small amount of collagen fiber group; A2 ~ F2 ROC curve of the validation set in a small amount of collagen fiber group

**Fig. 4:**
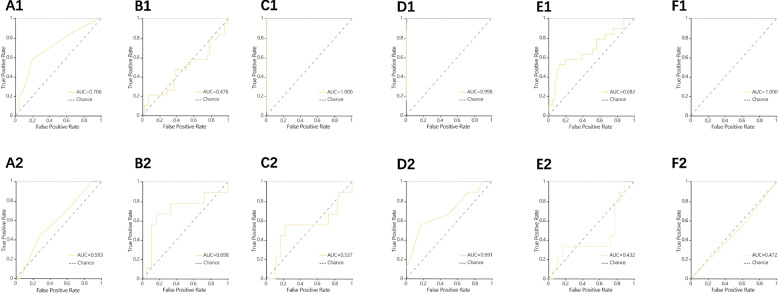
ROC curves of six methods in the Arterial phase (KNN, SVM, XGBoost, RF, LR and DT). A1 ~ F1 ROC curves for the training set in a small amount of collagen fiber group; A2 ~ F2 ROC curve of the validation set in a small amount of collagen fiber group

**Fig. 5:**
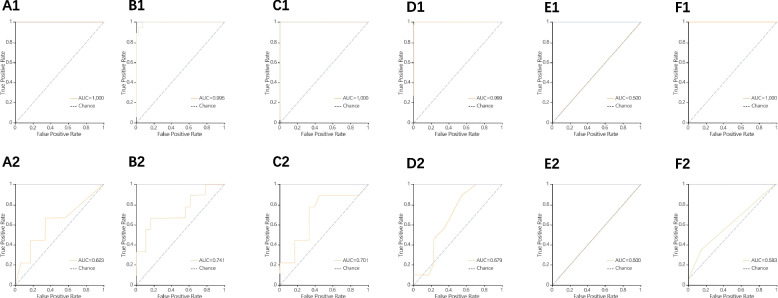
ROC curves of six methods in the venous phase (KNN, SVM, XGBoost, RF, LR and DT). A1 ~ F1 ROC curves for the training set in a small amount of collagen fiber group; A2 ~ F2 ROC curve of the validation set in a small amount of collagen fiber group

Figure 3 shows the classification results and the corresponding ROC curves of the six classifiers for the different feature sets extracted from the images. Compared to KNN, SVM, and LR classifiers, the RF, DT, and XGBoost classifiers trained with higher AUC values of 0.997, 1.0, and 1.0 for the training set, respectively. In the validation set, the highest AUC was found in the SVM classifier with a size of 0.722, while the RF, DT, and XGBoost classifiers also achieved good data with AUCs of 0.639, 0.694, and 0.639, respectively. In the dynamic and venous phase model comparison tests, the validation set AUCs were 0.698 and 0.741 for the SVM classifier, and the LR classifier The validation set AUCs were 0.432 and 0.500, respectively, so the AUCs of the models based on the venous phase was higher (Figs. 4 and 5).

We summarize the metrics of the six classifiers, precision, recall, f1 score, and support, in Tables 7 and 8 for the arterial and venous phases and all images, respectively. From the features extracted on all images, the more effective classifiers were RF, DT, and XGBoost, whose four categories of metrics in the training set were 0.90, 0.95, 0.92, 19, 1.00, 1.00, 1.00 19, and 1.00, 0.95, 0.97, 19, respectively. Except for this, the more effective classifiers for the model in both the arterial and venous phases were SVM.

**Table 7:**
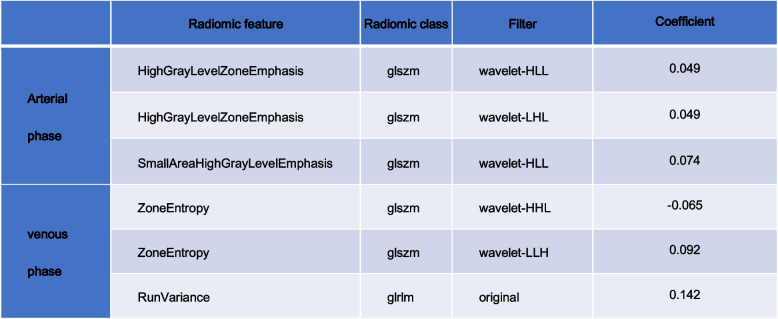
Description of the selected radiomics features with their associated feature group and filter

Label: GLSZM = Gray-Level Size Zone Matrix

Label: GLRLM = Gray Level Run Length Matrix, GLSZM = Gray-Level Size Zone Matrix

**Table 8:**
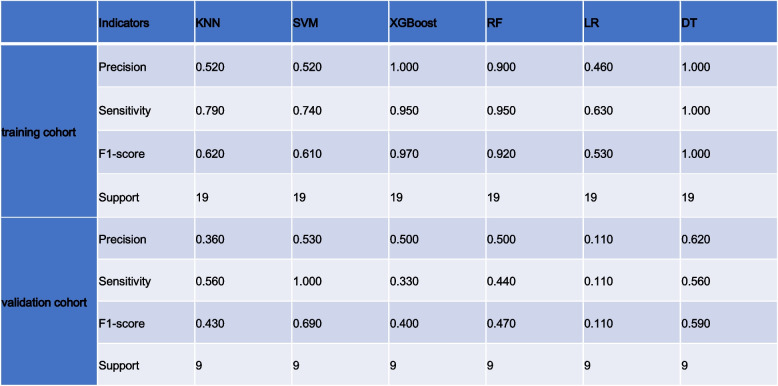
The results of Precision, Sensitivity, F1-score, and Support in training cohorts and validation cohorts

## Discussion

In this study, we developed a radiomics model from dual-energy CT Iodine map images for assessing collagen fiber content in the tumor microenvironment of kidney cancer. The results showed that there was an association between the radiomics features in renal dual-energy CT imaging and the collagen fiber content in the tumor microenvironment of kidney cancer, especially when trained using the SVM classifier, the model showed good predictive performance, and AUC is 0.722. When the arterial and venous phase model was compared, the venous phase model was more effective. Meanwhile, the clinical model developed in this study showed a significant correlation between collagen fiber content in the tumor microenvironment of kidney cancer and age and maximum tumor diameter.

In acquiring impact images of ccRCC patients, dual-energy CT was chosen for this study. Dual-energy CT takes advantage of the different X-ray attenuation values of different substances at different energies, such as iodine (Z = 53) and calcium (Z = 20), and acquires and analyzes images at different energies according to the different slopes of changes in attenuation values of these two substances. Currently, dual-energy CT has been applied in a large number of studies, and Han Bo [17] applied dual-source CT to the study of renal occupancies and found that dual-source CT increased the mean CT value. The results were in good agreement with pathology and significantly reduced radiation dose. Besides, in this study, the arterial and venous phase models were established separately and compared, and then it was concluded that the venous phase model has a higher AUC. Its corresponding classifier is more effective in the cases selected in this study. As a result, it is more convincing in assessing the collagen fiber content in the tumor microenvironment of kidney cancer.

In recent years, researchers have found in studies of liver, oesophageal, breast, and kidney cancers that radiomics has proven to be an effective tool for assessing information within the tumor microenvironment [18, 19]. At present, studies using imaging histology to assess the immune microenvironment and genes in kidney cancer have emerged. For example, Lianghong Jiao et al. [20] used imaging histology to explore the clinical and immune features of ccRCC associated with IL-23 expression levels and to develop a preoperative prediction model based on contrast CT scans. Based on the presence of differentially expressed metabolism-related prognostic genes and immune-related components, Wang Yi et al. [21] initially distinguished two distinct metabolic subtypes (C1 and C2 subtypes) and immune subtypes (I1 and I2 subtypes). Gao et al. [22] successfully classified patients into different subtypes based on gene expression levels in the tumor microenvironment (TME), and novel prognostic radiogenetics biomarkers correlated well with the immune-related gene expression status of ccRCC patients and could successfully stratify the survival status of patients in the TCGA database. However, it is rare to study the collagen fiber content of the tumor microenvironment using radiomics. Therefore, in this study, it is a valuable attempt for us that we used radiomics to assess the content of collagen fibers in the tumor microenvironment of kidney cancer and in the future, further evaluation of the complex information of the tumor microenvironment is more necessary to make better clinical decisions in the era of precision medicine.

This study assessed collagen fiber levels in the tumor microenvironment of kidney cancer using radiomics. Collagen is the most important component of the ECM and the most abundant protein in human tissues, with 28 unique isoforms having been identified [23–25]. Collagen fibers are located in the extracellular matrix and have important roles in tissue scaffolding, cell adhesion, cell migration, angiogenesis, tissue morphogenesis, and tissue recovery. Emerging collagen fibers can directly establish an invasive pathway for matrix metalloproteinase resistance to promote metastasis, and their density can facilitate macromolecular transport to alter renal cancer cell metabolism, inhibit transformed immune cell function, and promote gene expression [7]. In addition, collagen fibers stimulate fibroblast production and cross-linking between fibers, while at the same time increasing tissue fibrosis and stiffness to promote invasion and metastasis of kidney cancer cells. Also in the kidney, collagen plays a key function in branching morphogenesis, a process that involves the invasion of epithelial buds and tubes into the surrounding extracellular matrix-rich mesenchyme [26]. Therefore, preoperative assessment of collagen fibers in the tumor microenvironment of kidney cancer patients by non-invasive means is an important study for clinicians, and dual-energy CT IV radiomics by assessing and monitoring tumor characteristics (e.g., temporal and spatial heterogeneity) can achieve in-depth interpretation of information on the tumor microenvironment of kidney cancer and be used for clinical diagnosis and prognosis.

There are some limitations of the study. First of all, since this is a single-center study with a small sample size, more research is required to assess the model and findings using a larger data set. Second, rather than three-dimensional CT scans, the radiomics model uses two-dimensional images, necessitating further evaluation of its performance using three-dimensional data. Additionally, user dependence and variability may be introduced since tumor segmentation is done manually. In future work, fully automated segmentation may become a reality. Finally, this work is a retrospective study, so selection bias cannot be completely avoided.

## Conclusion

In conclusion, preoperative models based on radiomics features of dual-energy CT IV can predict collagen fiber content in the tumor microenvironment of kidney cancer, Radiomics models, as opposed to the conventional visual evaluation of images, can provide a tool to help assessing the collagen fiber content, better informing clinical prognosis and patient management. Further evaluation of our findings on a large dataset will be necessary for future work.

## Data Availability

The datasets used and/or analyzed during the current study are available from the corresponding author on reasonable request.
